# Nutrition for Training and Performance

**DOI:** 10.1007/s40279-014-0262-y

**Published:** 2014-10-30

**Authors:** Lawrence L. Spriet

**Affiliations:** Department of Human Health and Nutritional Sciences, University of Guelph, Room 354, ANNU Building, Guelph, ON N1G 2W1 Canada

The human body is designed for movement and capable of incredible athletic and sporting achievements. Attempts to improve the ability to perform on the athletic stage are never-ending and involve a serious dedication to training. Athletes, coaches, support personnel, and scientists are constantly experimenting with new ways to improve training and its applicability to performance. Nutrition is clearly a major factor in the success of training and performance as fuel is needed to power the human engine. The amount, type, and timing of nutritional intake play a large role in the physical and mental success of the athlete. In addition, nutritional intake also influences the adaptation to training and the recovery from training, to positively impact performance.

The Gatorade Sports Science Institute (GSSI) brought together researchers for a meeting in February 2013 to discuss the recent evidence that nutrition influences athletic training and performance. Following the meeting, the authors were asked to summarize the recent work in their research area, resulting in the manuscripts in this Sports Medicine supplement.

A major step forward in sports nutrition has been the ability to more thoroughly understand how training alters the body at the molecular level, in both acute and chronic situations. Various forms of training have been shown to activate cell signalling pathways that ultimately produce protein-specific adaptations geared to improving sport-specific performance. Whether engaging in classic moderate intensity–high volume endurance training, high intensity intermittent–low volume training, or resistance training, the body remodels to more aptly handle the specific physical stress. Nutrition is at the core of these adaptations through the provision of fuel and by shaping the adaptations. Concurrent training has long been of interest, where both resistance and endurance training sessions are completed over the course of a week or even on the same day. Engaging in high intensity intermittent–low volume training as compared with the classic moderate intensity–high volume endurance training has also been a central theme in the past few years. What can be achieved in these situations? Does the stimulus from one form of training dominate the other—and is one form of training more time efficient than the other? A related issue, especially in the case of high-end athletes, is monitoring training load and understanding when an athlete is overloaded and likely to underperform. What are the measures that athletes, trainers, therapists, coaches, nutritionists, and physiologists can use to accurately assess the situation? On the other side of the movement spectrum, what happens when muscles are not used either through injury or a sedentary lifestyle? Can nutrition in the form of antioxidants prevent the negative consequences associated with these situations?

Serious athletes do all they can to maximize their physical and mental training, nutrition, recovery, and sleep quality. Once this has been achieved, are there nutritional means to acutely and chronically improve their training and performance with supplements like caffeine, creatine and buffering agents? In certain sports and athletic endeavours, losing a small amount of body mass without compromising training and performance can lead to improved performance. Can manipulating dietary protein intake aid in this pursuit and has this been thoroughly studied? Lastly, it is clear that athleticism and the pursuit of training and performance is no longer solely the domain of the young. The number of older athletes is on the rise and the phrase, “60 is the new 45” is commonly heard among active older individuals. What are the nutritional needs of this group of people and what needs to be studied to answer this question?

The role of sports nutrition and exercise is becoming increasingly linked with health and performance and understanding the interactions between nutrition and exercise will help us to provide accurate information to exercisers and athletes.

